# POEMS syndrome and calciphylaxis: an unrecognized cause of abnormal small vessel calcification

**DOI:** 10.1186/s13023-016-0421-3

**Published:** 2016-04-12

**Authors:** Nobuyuki Araki, Sonoko Misawa, Kazumoto Shibuya, Satoshi Ota, Takashi Oide, Asuka Kawano, Minako Beppu, Yukio Nakatani, Satoshi Kuwabara

**Affiliations:** Department of Neurology, Graduate School of Medicine, Chiba University, 1-8-1 Inohana, Chuo-ku, Chiba 260-8670 Japan; Department of Pathology, Chiba University Hospital, 1-8-1 Inohana, Chuo-ku, Chiba 260-8677 Japan; Diagnostic Pathology, Graduate School of Medicine, Chiba University, 1-8-1 Inohana, Chuo-ku, Chiba 260-8670 Japan

**Keywords:** Calciphylaxis, POEMS syndrome, Vascular calcification, Interleukin-6, Vascular endothelial growth factor

## Abstract

**Background:**

Calciphylaxis is a syndrome consisting of vascular calcification, thrombosis, and skin necrosis. The syndrome develops often in chronic hemodialysis patients. However, there have been several case reports on calciphylaxis in patients with POEMS (polyneuropathy, organomegaly, endocrinopathy, M-protein, and skin changes) syndrome, a systemic disease associated with plasma cell dyscrasia and upregulation of vascular endothelial growth factor (VEGF).

**Methods:**

In 76 POEMS patients and 86 age- and gender-matched disease controls, we studied abnormal small vessel calcification by computed tomography (CT) of the soft tissues. Clinical and laboratory profiles were compared between POEMS patients with and without calciphylaxis. Histological examination was performed in six autopsy cases.

**Results:**

Small vessel calcification on CT was found in 17 % of POEMS patients and in none of the controls (*P* < 0.001). Autopsy confirmed calciphylaxis in 2 (33 %) patients. Among POEMS patients, higher disease activity, including more severe neuropathy and ascites, higher serum levels of interleukin-6, and lower serum albumin levels, was associated with the development of calciphylaxis. Serum levels of creatinine, calcium, and phosphate were not related to the presence of calciphylaxis.

**Conclusions:**

Calciphylaxis is often present in patients with POEMS syndrome. Upregulation of multiple inflammatory cytokines such as VEGF and interleukin-6 may contribute to the development of calciphylaxis, by entirely different mechanism from that in chronic dialysis. POEMS syndrome should be recognized as a potential cause of calciphylaxis.

## Background

Calciphylaxis is a rare and life-threatening disease characterized by calcification of the small- to medium-sized arteries in the dermis and subcutis with intimal fibrosis, resulting in progressive cutaneous necrosis accompanied by severe pain [1–4]. End-stage renal disease and resulting hyperparathyroidism have been considered as major causes for calciphylaxis, which occurs in approximately 4 % of patients on chronic hemodialysis [4]. Calciphylaxis has also been reported in non-uremic patients associated with the use of warfarin, alcoholic liver cirrhosis, obesity, chronic inflammation, and prolonged corticosteroid administration [5–10]. The treatment of calciphylaxis requires a multidisciplinary approach involving optimal wound management, antibiotic use, and correction of biochemical abnormalities [4, 11, 12], but calciphylaxis is a fatal in 60–80 % of cases due to bacterial infection [1, 2], and therefore early diagnosis and treatment are necessary.

Polyneuropathy, organomegaly, endocrinopathy, M-protein, and skin changes (POEMS) syndrome is a rare systemic disease characterized by a monoclonal plasma cell proliferation and multiorgan involvement [13, 14]. Markedly upregulated vascular endothelial growth factor (VEGF), presumably produced by plasma cells, has been implicated in the pathogenesis of POEMS syndrome [13, 14]. As included in the disease acronym, skin changes are one of the major features of the disorder. Typical skin changes include hyperpigmentation, acrocyanosis, hemangioma, telangiectasia, hypertrichosis, and skin thickening [14]. To date, five cases of POEMS syndrome have been reported to present calciphylaxis [15–19]. We experienced a POEMS syndrome patient who developed severe calciphylaxis, and this led us to systematically investigate the prevalence of calciphylaxis and its risk factors in POEMS syndrome.

### Case report

**Fig. 1:**
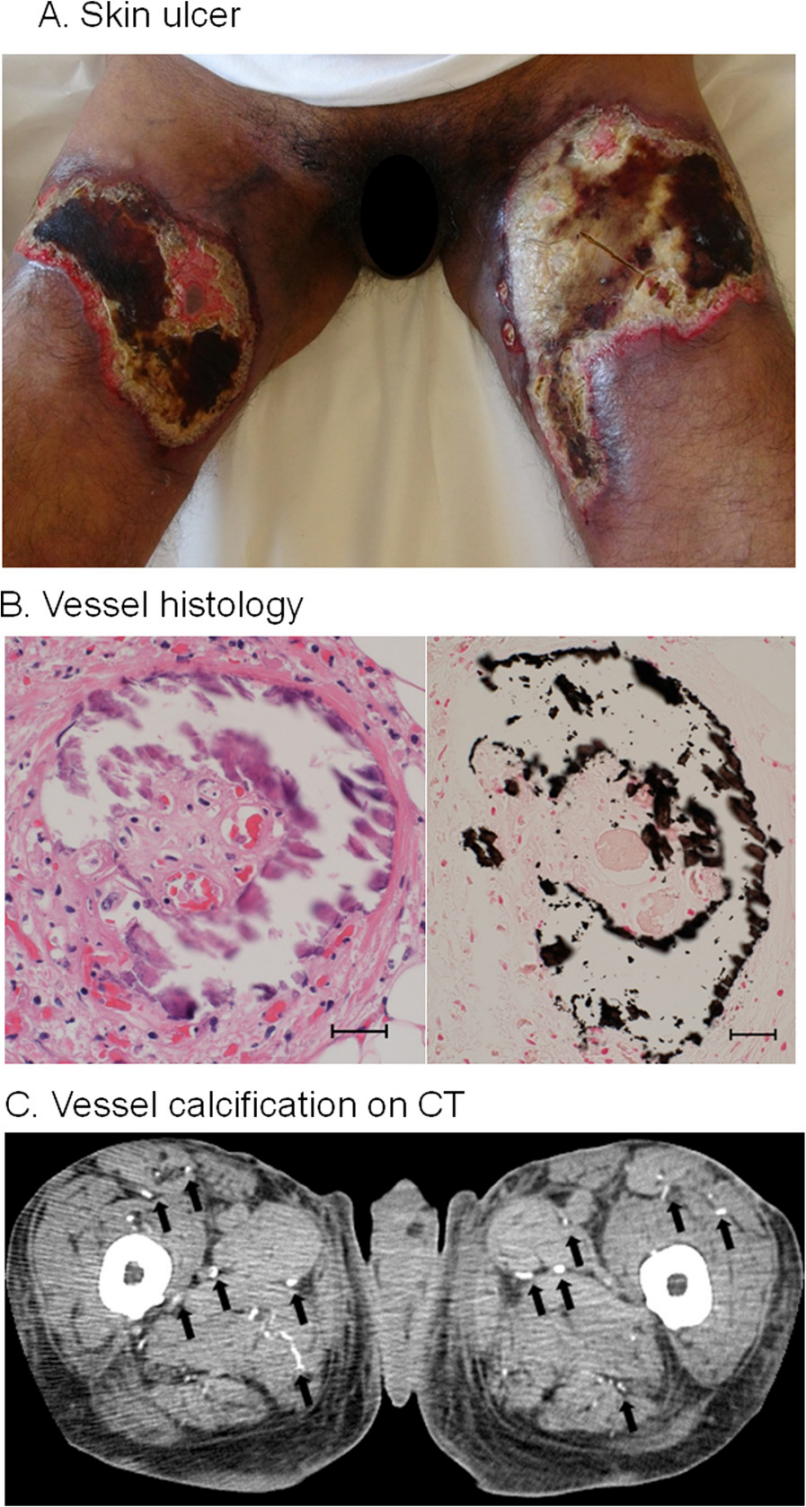
Findings in a patient with severe skin ulcers secondary to calciphylaxis. **a** Calciphylaxis-induced skin ulcers on admission. **b** Histology of biopsy specimen from the ulcer on the left thigh stained with hematoxylin and eosin stain (B1) and von Kossa stain (B2); Scale bars indicate 100 μm. Calcium deposits in the media of small- and medium-sized arteries. **c** Plain computed tomography showed multiple subcutaneous and intramuscular calcifications in the soft tissue of the thighs

A 62-year-old male with a six-year history of POEMS syndrome was admitted to our hospital because of painful skin ulcers on both thighs. The diagnosis of POEMS syndrome had been made based on polyneuropathy, M-proteinemia (IgA λ type), elevated serum VEGF levels (4810 pg/ml; normal <1000 pg/ml), edema, ascites/pleural effusion, gynecomastia, and skin pigmentation. Treatment with corticosteroids and thalidomide followed by peripheral blood stem cell transplantation (PBSCT) had resulted in clinical remission, but 2 years later he had a relapse with skin ulcers. On admission, severe skin ulcers were present on both thighs (Fig. 1a). Thalidomide was switched to lenalidomide, but the ulcers continued to increase in size. Laboratory examination showed slight leukocytosis and hypoalbuminemia with a high serum C-reactive protein level. Serum calcium and phosphorus concentrations and parathyroid hormone levels were normal. Histology of hematoxylin and eosin (HE) and von Kossa stained sections from the ulcerative skin showed calcified vessels in the deep dermis (Fig. 1b). Computed tomography (CT) revealed abnormal calcification of the small vessels in the soft tissues of the hip/thigh (Fig. 1c). Based on these findings, a diagnosis of calciphylaxis was made. The ulcers enlarged further over the next 8 weeks. He was treated with continuous hemodiafiltration and high-dose corticosteroids because of septic shock. After transplantation of skin grafts, the ulcers gradually improved over the next 12 weeks.

## Methods

### Subjects

We reviewed the records of 76 patients with POEMS syndrome (52 men), seen at Chiba University Hospital between 1992 and 2013. Patient age ranged from 31 to 77 years (median, 56 years). All patients fulfilled the diagnostic criteria of POEMS syndrome [13, 20]. One patient with POEMS syndrome receiving chronic hemodialysis was excluded, and the data from the remaining 75 patients were analyzed.

To determine whether the vessel changes were caused by calciphylaxis and not by aging, we reviewed the records of 86 age-matched patients (63 men) with various neurological diseases who underwent CT evaluation during the same period. The disorders included Parkinsonism (Parkinson disease, multiple system atrophy, dementia with Lewy bodies, progressive supranuclear palsy, and cortico-basal syndrome; *n* = 9), amyotrophic lateral sclerosis (*n* = 5), other neurodegenerative diseases (*n* = 7), multiple sclerosis (*n* = 4), immune-mediated neuropathies (*n* = 13), and others (*n* = 42). The study protocol was approved by the Ethics Committee of the Chiba University School of Medicine, Chiba, Japan.

### Diagnosis of calciphylaxis

Diagnostic criteria for calciphylaxis have not been established [11]. Therefore, we defined probable calciphylaxis as (1) livedo reticularis that was followed by skin ulceration covered with necrotic tissue, or (2) calcification of small- and medium-sized vessels on CT or histopathology. CT images from the top of the chest to the upper thigh were reviewed for the presence of calcification of small- and medium-sized vessels (diameter <2 mm) in the soft tissues of the hip/thigh. Calcification of small-sized vessels appeared to indicate calciphylaxis [21], as shown in the patient presented above.

### Clinical and laboratory evaluation

We investigated the presence of ulcers and vessel calcification on CT, and vascular risk factors such as diabetes, hypertension, and hyperlipidemia. We then compared clinical and laboratory data and treatment history between POEMS syndrome patients with probable calciphylaxis and those without it. The overall neuropathy limitation scale (ONLS) was used to evaluate the severity of neuropathy. The presence of pleural effusion/ascites on CT was evaluated and history of events suggestive of thrombosis, such as cerebral infarction, myocardial infarction, and pulmonary thromboembolism, was also compared between POEMS patients with and without probable calciphylaxis. Serum concentrations of VEGF, tumor necrosis factor-α (TNF-α), interleukin-6 (IL-6), creatinine, calcium, phosphate, and albumin, and values of platelet count, prothrombin time-international normalized ratio (PT-INR), activated partial thromboplastin time (APTT), fibrinogen degradation products (FDP), and D-dimer measured around the time of CT (within 4 weeks) were recorded. Corrected serum concentration of calcium ([Ca]) which was calculated by formula showed below, were used for statistical analysis [22].

$$ \mathrm{Corrected}\ \left[\mathrm{C}\mathrm{a}\right]\ \left(\mathrm{mg}/\mathrm{dl}\right) = \mathrm{Measured}\ \left[\mathrm{C}\mathrm{a}\right]\ \left(\mathrm{mg}/\mathrm{dl}\right) + \left\{4.0\hbox{-} \left[\mathrm{Alb}\right]\ \left(\mathrm{g}/\mathrm{dl}\right)\right\} $$

$$ \left[\mathrm{C}\mathrm{a}\right]:\ \mathrm{serum}\ \mathrm{concentration}\ \mathrm{of}\ \mathrm{calcium},\ \left[\mathrm{Alb}\right]:\ \mathrm{serum}\ \mathrm{concentration}\ \mathrm{of}\ \mathrm{a}\mathrm{lbumin} $$

### Autopsy cases

During the study period, six of the 76 patients with POEMS syndrome died. Autopsy reports were reviewed and abdominal skin specimens from four patients were obtained and investigated histopathologically by HE and von Kossa staining. In the remaining two patients skin specimens were not taken. The skin specimens from three autopsy cases of plasma cell myeloma were compared as negative controls.

### Statistical analysis

All statistical analyses were performed using SPSS statistic software (IBM, version 20). The differences in gender, prevalence of diabetes, hypertension, and hyperlipidemia between the POEMS syndrome group and the neurological disease control group were compared using Pearson’s chi-squared test. The differences in age and serum concentration of creatinine were compared using the Mann–Whitney *U* test and the differences in the prevalence of skin ulcers and calcification seen on CT were compared using Fisher’s exact test.

The differences in gender, ONLS score, presence of ascites, history of thrombosis, and treatments between the patient groups were compared using Fisher’s exact test. The differences in the presence of pleural effusion and history of steroid therapy were compared using Pearson’s chi-squared test and the differences in age, disease duration, and laboratory values were compared using the Mann–Whitney *U* test.

## Results

### The frequency of calciphylaxis

**Fig. 2:**
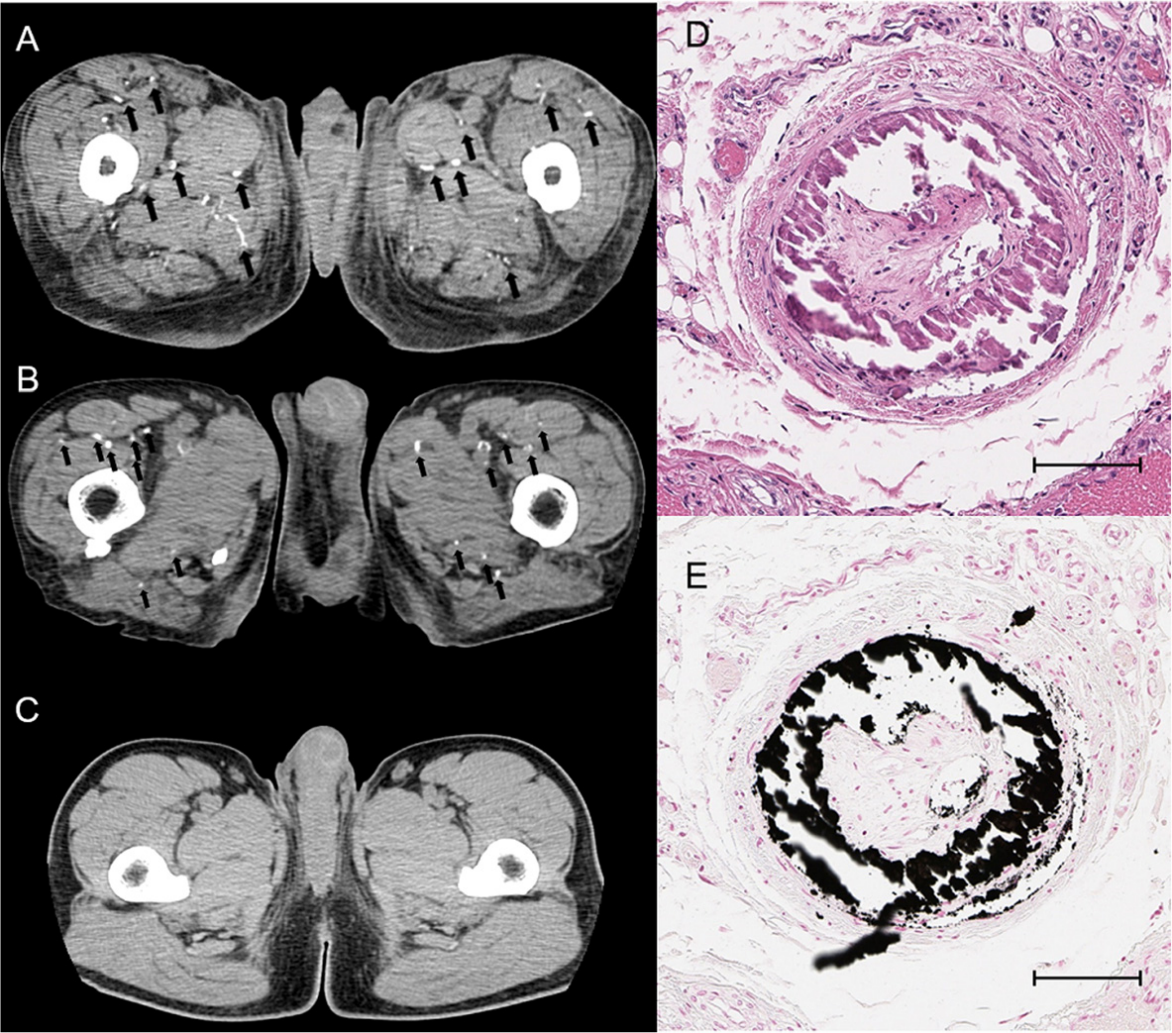
Findings of calcification on computed tomography (CT). **a** Plain CT of a POEMS syndrome patient with calciphylaxis. **b** The same CT changes in a chronic hemodialysis patient. **c** CT of a POEMS syndrome patient with no calcifications. **d** Ring-like calcification and occlusion of subcutaneous arterioles of an autopsy case. Autopsy case skin samples were obtained from a grossly normal lesion. Medial thickening and intimal loose fibrous proliferation caused arteriolar occlusion without any inflammation as seen on hematoxylin and eosin staining. **e** Calcification in the tunica media of arterioles as seen with von Kossa staining; Scale bars indicate 100 μm

The prevalence of calciphylaxis in POEMS syndrome patients and the neurological disease controls is shown in Table 1. Skin ulcers consistent with calciphylaxis features were present in 4 % of POEMS patients and in none of the controls. Small vessel calcification on CT was observed in 17 % of POEMS patients, and in none of the controls (*P* = 0.00079), indicating that calciphylaxis was not caused merely by aging, but was specifically present in patients with POEMS syndrome (Fig. 2). There were no significant differences in the prevalence of diabetes, hypertension, or hyperlipidemia between the two groups. Calcification of the large arteries such as the aorta (84 % in POEMS syndrome patients versus 76 % in controls; *P* = 0.19) and coronary arteries (31 % in POEMS syndrome patients versus 33 % in controls; *P* = 0.80) were similarly present in both the groups, presumably reflecting the effects of aging and atherosclerosis.

**Table 1:** Vascular calcification and risk factors

	POEMS syndrome	Disease control	*P*-value
	(*n* = 75)	(*n* = 86)	
Gender; men:women	52:23	62:24	0.70
Age (years); mean (SD)	57 (11)	59 (14)	0.19
Calciphylaxis			
Skin ulcer (%)	4 %	0 %	0.097
CT^a^	17 %	0 %	0.00079
Vascular risk factor			
Diabetes	19 %	20 %	0.86
Hypertension	33 %	29 %	0.56
Hyperlipidemia	31 %	45 %	0.056

Disease controls include patients with various neurodegenerative or inflammatory diseases (see text)

^a^Small vessel calcification in the soft tissue of the hip/thigh

### Histology in autopsy cases

Of the four autopsy cases which had obtained skin graft, two patients with no history of dialysis showed calcification in vessels with a diameter <0.6 mm in the abdominal wall skin (Fig. 2d, e). No significant histopathological findings were obtained from skin specimens of the three plasma cell myeloma autopsy cases. The findings in the POEMS syndrome cases were consistent with calciphylaxis, and small vascular calcification was also present on CT of the soft tissue in the hip/thigh in these two patients.

### Factors associated with probable calciphylaxis in POEMS syndrome

Clinical and laboratory findings were compared between the 13 POEMS patients with and the 62 without calciphylaxis (Table 2). Compared with the group without calciphylaxis, the calciphylaxis group included a higher proportion of men, and the patients showed more severe neuropathy (higher ONLS score) or ascites. In patients with calciphylaxis, serum levels of IL-6 were significantly higher compared with patients without calciphylaxis, but serum VEGF levels were not significantly different between the two groups. Serum albumin concentration was significantly lower in the calciphylaxis group compared with the group without it.

**Table 2:** Clinical and laboratory profiles of POEMS Syndrome Patients with and without calciphylaxis

	Calciphylaxis		*P*-value
	Present (*n* = 13)	Absent (*n* = 62)	
Gender; men:women	12:1	40:22	0.043*
Age (years); mean (SD)	58 (11)	57 (11)	0.89
Disease duration	3.1 (2.0)	2.9 (3.3)	0.21
ONLS Leg score >5	46 %	18 %	0.042*
Pleural effusion	54 %	40 %	0.28
Ascites	54 %	13 %	0.0028*
Laboratory data			
VEGF (pg/ml)	4100 (3100)	5100 (5500) (*n* = 57)	1.0
TNF-α (pg/ml)	18 (24) (*n* = 7)	8.9 (11) (*n* = 17)	0.22
IL-6 (pg/ml)	12 (4.4) (*n* = 5)	4.4 (2.1) (*n* = 13)	0.0013*
Cre (mg/dl)	0.86 (0.45)	0.94 (0.65)	0.83
Ca (mg/dl)	9.0 (0.48)	8.8 (0.42)	0.19
I-P (mg/dl)	4.1 (0.98) (*n* = 10)	4.0 (1.2) (*n* = 54)	0.88
Ca × I-P (mg^2^/dl^2^)	36 (8.4) (*n* = 10)	36 (9.9) (*n* = 54)	0.89
Albumin (g/dl)	3.1 (0.44)	3.7 (0.54)	0.00035*
Plt (10^3^/μl)	260 (130)	310 (160)	0.21
Treatment			
Corticosteroid	62 %	53 %	0.58
Thalidomide	15 %	8 %	0.35
Melphalan	8 %	19 %	0.29
PBSCT	15 %	3 %	0.14

Data are shown as mean (SD). ONLS, Overall Neuropathy Limitation Scale; VEGF, vascular endothelial growth factor; TNF-α: tumor necrosis factor-α; IL-6: Interleukin-6;

Ca, serum concentration of calcium corrected by albumin concentration; I-P, serum concentration of phosphate; Plt, platelet count; PBSCT: peripheral blood stem cell transplantation

*: p<0.05

There were no significant differences between the groups for serum concentrations of creatinine, calcium, or phosphate, which are known risk factors for calciphylaxis in patients undergoing chronic hemodialysis. POEMS patients with and without calciphylaxis received similar treatments. There were also no significant difference between the groups for hypertension (38 % in POEMS syndrome with calciphylaxis versus 32 % in POEMS syndrome without calciphylaxis; *P* = 0.45), diabetes (15 % in POEMS syndrome with calciphylaxis versus 19 % in POEMS syndrome without calciphylaxis; *P* = 0.55) and hyperlipidemia (38 % in POEMS syndrome with calciphylaxis versus 29 % in POEMS syndrome without calciphylaxis; *P* = 0.36) as a risk factor of atherosclerosis.

## Discussion

Our results show that calciphylaxis is not rare, and probable calciphylaxis, which fulfil both skin feature and vessel calcification, occurs in 4 % of patients with POEMS syndrome, and calciphylaxis like calcification on CT were seen 17 % of them. Because the resolution of CT is not sufficiently high to detect very small vessel calcification, the prevalence might be underestimated, because two of the four autopsy cases had histological evidence of calciphylaxis. Calcification of large vessels such as the aorta and coronary arteries on CT, presumably caused by aging and atherosclerosis, was similarly seen in POEMS patients and disease controls, but calciphylaxis was specifically observed in patients with POEMS syndrome, suggesting that the pathophysiology of the syndrome is directly related to the development of calciphylaxis.

Vascular calcifications are classified into arterial intima calcification (AIC) and arterial media calcification (AMC), or Mönckeberg arteriosclerosis [23, 24]. AIC represents an advanced atherosclerosis associated with the development of plaques and occlusive lesions. In contrast, AMC is commonly associated with end-stage renal disease and diabetes [23], and observed primarily in muscle-type conduit arteries, such as the femoral and tibial arteries. AIC and AMC showed different patterns of vessel calcification on X-ray [25], with discrete, irregular, and patchy distribution of calcification typical of AIC, whereas uniform linear calcification suggests AMC.

Pathologically, the specific feature of calciphylaxis is medial calcification in small-sized vessels with a diameter <0.6 mm [4]. It is difficult to detect calcification of small-sized vessels on CT, but previous reports have shown that calciphylaxis patients often have abnormal vascular calcification in muscle and connective tissue in the lateral thighs, which can be detected by X-ray and CT [16, 21]. Our study showed that the distribution of calcification in POEMS syndrome resembles that for AMC, and suggested that calcification of vessels <2 mm in diameter detected on CT would represent calciphylaxis. In the case presented above, histopathology with von Kossa staining identified small vessel calcification consistent with calciphylaxis. Moreover, the two autopsy cases showed small vessel calcification on both CT and histology. Based on these findings, it is likely that vascular calcification in patients with POEMS syndrome indicates calciphylaxis.

The precise mechanisms for AMC in POEMS syndrome remain unknown. Previous studies have suggested that the final common pathway that leads to AMC is nuclear factor κ-B (NFκB) activation [3]. NFκB is a key transcription factor for numerous cellular functions, including production of several growth factors, inflammatory mediators, adhesion molecules, and cytokines, and it regulates the balance between bone deposition and resorption. Activation of NFκB results in osseous mineral resorption and extraosseous mineral deposition, causes osteoblastic transformation in vascular smooth muscle cells, and finally leads to AMC. Receptor activator of NFκB ligand (RANKL) and its antagonist, osteoprotegerin (OPG), play a critical role in bone remodeling [26].

Multiple proinflammatory cytokines such as interleukin-1, TNF-α, and IL-6 are upregulated in POEMS syndrome [14, 27, 28]. IL-6 may contribute to vascular calcification by activating RANKL [29]. Our results show that, in POEMS patients with calciphylaxis IL-6 levels were significantly higher compared with POEMS patients without calciphylaxis. We speculate that a upregulated cytokines may induce vascular calcification in POEMS syndrome.

There are several limitations in this study. First, small vessel calcification on CT is not a direct evidence for calciphylaxis. However, we confirmed a relationship of the CT findings and histological evidence of calciphylaxis in patients with biopsy or autopsy, and believe that the CT findings reflect the presence of calciphylaxis. Second, some medications may affect the finfings; for example the use of statins that have anti-inflammatory effects was not strictly checked in both the POEMS and control groups. However, we think that this factor did not largely affect the findings, and that calciphylaxis develops by POEMS syndrome itself.

## Conclusion

Comparison between POEMS syndrome patients with and without calciphylaxis like calcification showed that more severe neuropathy (higher ONLS score), more ascites, and lower serum concentrations of albumin were associated with the presence of calciphylaxis like calcification, suggesting that it is likely to develop with higher disease activity. On the other hand, known risk factors for calciphylaxis in hemodialysis, such as high serum calcium and phosphate levels, had no relationship to calcification in POEMS syndrome patients, indicating that the mechanisms for calciphylaxis are different between chronic hemodialysis and POEMS syndrome. We suggest that POEMS syndrome should be recognized as a cause of calciphylaxis, and that patients will benefit from appropriate and timely management.

## Abbreviations

AIC: Arterial intima calcification
AMC: Arterial media calcification
APTT: Activated partial thromboplastin time
BMP: Bone morphogenetic protein
CHDF: Continuous hemodiafiltration
IL-6: Interleukin-6
NFκB: Nuclear factor κ-B
ONLS: Overall neuropathy limitation scale
PBSCT: Peripheral blood stem cell transplantation
POEMS: Polyneuropathy, organomegaly, endocrinopathy, M-protein, and skin changes
RANKL: Receptor activator of NFκB ligand
TNF-α: Tumor necrosis factor-α
VEGF: Vascular endothelial growth factor
